# Mechanism of Catechol-O-methyltransferase Regulating Orofacial Pain Induced by Tooth Movement

**DOI:** 10.1155/2021/4229491

**Published:** 2021-10-23

**Authors:** Yonglong Zhou, Zhiping Song, Shibiao Chen, Fen Yao, Jian Liu, Zhiqiang Ouyang, Zhengyu Liao

**Affiliations:** ^1^Department of Orthodontics, School of Stomatology, The Key Laboratory of Oral Biomedicine, Affliated Stomatological Hospital of Nanchang University, No. 49 Fuzhou Road, Nanchang, 330006 Jiangxi Province, China; ^2^Department of Anesthesia, The First Affiliated Hospital of Nanchang University, No. 17 Yongwaizheng Street, Nanchang, 330006 Jiangxi Province, China

## Abstract

**Objective:**

To explore the mechanism of catechol-O-methyltransferase (COMT) in tooth movement pain.

**Methods:**

The experimental groups were randomly allocated into the healthy control, sham operation, model, model+shCOMT experimental, model+shCOMT control, and model+COMT antagonist groups. A tooth movement pain model was established. The pain stimulation and behavior test were performed. The duration of grooming behavior was determined. The appropriate experimental force and duration for application were selected. COMT shRNA vector was constructed and packaged as adenovirus. The shCOMT adenovirus was injected into the left infraorbital foramen. Seven days later, the trigeminal ganglia of all treatment groups were obtained. The COMT and IL-17 expressions were detected by western blot. The appropriate COMT antagonist concentration was selected. The pathological results of each group were detected by HE staining. The tooth movement distance was determined. The COMT gene expression was detected by FISH. The COMT and IL-17 expressions in the right trigeminal ganglion tissue of each group were detected by western blot.

**Results:**

The 60 g force and 14-day duration required the lowest stimulus intensity, the duration of grooming behavior was the longest, and the effect on COMT and IL-17 was the most significant. In the model group, formation of digestive cavity was seen in the trigeminal ganglion tissue, with infiltration of inflammatory cells, upregulation of the COMT and IL-17 expressions, and significant increase in the tooth movement distance. Compared with the model group, the shCOMT experimental group and the COMT antagonist group significantly improved the trigeminal ganglion tissue injury, significantly decreased the tooth movement distance, and significantly inhibited the COMT and IL-17 expressions.

**Conclusion:**

The efficiency of tooth movement can be influenced by interfering the COMT-related gene expression. This proves that the COMT system can regulate the orthodontic tooth movement pain.

## 1. Introduction

Malocclusion has become the third largest oral clinical disease after dental caries and periodontal disease. Oral epidemiological survey shows that the overall prevalence rate of malocclusion among Chinese residents is 67.82% [1]. With the progress of society and economic development, more and more people are or will be undergoing orthodontic treatment. However, it needs to apply certain correction force to the misaligned teeth, dental arch, or jaw, and the whole process may cause pain [2]. In current clinical studies, there is no proper method to control pain caused by orthodontic force stimulation. Although nonsteroidal anti-inflammatory drugs (NSAIDs) can reduce the inflammatory reaction in pulp and periodontal tissue after tooth movement by inhibiting the release of prostaglandins and relieve pain, they also inhibit the cyclooxygenase activity and thus reduce the rate of tooth movement. Thus, they are not suitable for orthodontic pain control [3, 4]. Therefore, elucidating the underlying neural mechanisms is essential for improving orthodontic pain treatment.

Catechol O-methyl-transferase (COMT) is an important type of phase II metabolic enzyme. It combines with S-adenosylmethionine (SAM or AdoMet) and then with the substrate to transfer the methyl group of S-adenosylmethionine to the hydroxyl group of the catechol substrate to form hydroxymethyl products [5]. Studies found that COMT is mainly distributed in postsynaptic neurons and glial cells, as well as in peripheral tissues [6]. It can participate in the regulation of dopamine and epinephrine/norepinephrine neurotransmitters closely related to pain [7]. Statistics found that COMT gene of patients with resistance disease is positively correlated with the intensity of oral pain and analgesic requirements [8]. In adults, there is also a correlation between bruxism and COMT, and orthodontic treatment is also related to it, but there is no relevant experimental evidence that directly prove the therapeutic effect of COMT on orthodontic tooth movement.

IL-17 was involved in the regulation of orthodontic tooth movement in different periodontal conditions. Orthodontic force caused limited increase of IL-17 mRNA expression, and the jiggling forces might exacerbate OIRR compared with heavy forces, as evidenced by the increased expression of IL-17 and IL-34 in odontoclasts obtained from resorbed roots [9, 10]. Ha et al. found that [11] COMT is closely associated with the expression of IL-1*β* and IL-6 on the anti-inflammatory activity of luteolin metabolites. However, there is no relevant literature report on the relationship between COMT and IL-17.

The orthodontic tooth movement model is the experimental basis for studies on mechanism and treatment. However, there is no reliable behavioral evaluation method for the establishment of orthodontic tooth movement model, and the setting of orthodontic force in the previous studies results is mostly inconsistent [12, 13]. Therefore, this study is carried out to improve the behavioral evaluation model of experimental tooth movement pain in rats, screen the best shRNA sequence to silence COMT, and interfere with the related gene expressions, so as to explore the regulatory effect of COMT system on orthodontic tooth movement pain and the relationship between the COMT and IL-17.

## 2. Materials and Methods

### 2.1. Experimental Animals

Sixty-seven male 8-week-old Sprague Dawley (SD) rats were purchased from Hunan SJA Laboratory Animal Co., Ltd., Hunan, China (license number SCXK (Xiang) 2019-0004). The rats were raised in a nontoxic plastic rat box, stainless steel wire cage cover, with metal cage frame. The cage was generally movable and could withstand various methods of sterilization. This study was approved by the Animal Use and Care Committee of the Affiliated Stomatological Hospital of Nanchang University (permit no. 2017007) and was conducted in accordance with the principles and procedures outlined in the National Institutes of Health (NIH) Guide for the Care and Use of Laboratory Animals (NIH, revised 2011). This study was reported in accordance with the ARRIVE guidelines (Animals in Research: Reporting In Vivo Experiments, Kilkenny, Browne, Cuthill, Emerson, & Altman, 2010).

### 2.2. Main Reagents and Instruments

COMT antagonist (Entacapone) (Glpbio Technology Inc., Montclair, CA, USA), polyvinylidene difluoride (PVDF) membrane (Millipore Sigma, Billerica, MA, USA), enhanced chemiluminescence (ECL) Plus (Thermo Fisher Scientific Inc., Waltham, MA, USA), mouse monoclonal anti-glyceraldehyde 3-phosphate dehydrogenase (GAPDH) (1 : 2000), goat anti-mouse immunoglobulin G (IgG) heavy and light chain (H+L) horseradish peroxidase (HRP) conjugate (1 : 2000) (ZSGB-Bio, Beijing, China), rabbit anti-catechol-O-methyltransferase (COMT) (1 : 500-1000) (Proteintech Group, Inc., Rosemont, IL, USA), rabbit anti interleukin- (IL-) 17 (1/500-2000) (Bioss Inc., Woburn, MA, USA), r-COMT short hairpin RNA (shRNA) adenovirus (ZHBY Biotech Co., Ltd., Nanjing, Jiangsu, China), COMT RNA fluorescence in situ hybridization (FISH) probe (Synbio Tech Inc., Kaohsiung, Taiwan), TRIzon reagent, ultrapure RNA extraction kit (Beijing Cowinbioscience Co., Ltd., Beijing, China); HiScript II Q reverse transcriptase (RT) SuperMix for qPCR (+gDNA wiper) (Vazyme Biotech Co., Ltd., Nanjing, Jiangsu, China), Synergy Brands, Inc. (SYBR®) Green polymerase chain reaction (PCR) Master Mix (Xiamen Life-Int Technology Co., Ltd., Xiamen, Fujian, China), orthodontic spring (length 9 mm, Shinye Orthodontic Products Co., Ltd. Hangzhou, Zhejiang, China), orthodontic ligature wire (round, wire diameter 0.2 mm, Hangzhou Aosu Medical Devicement Co., Ltd., Hangzhou, Zhejiang, China), mini handheld skull drill-national standard (78001, RWD Life Science Co., Ltd., Shenzhen, Guangdong, China), vertical protein electrophoresis system (DYY-6C, Beijing Liuyi Biotechnology Co., Ltd., Beijing, China, ultra-sensitive chemiluminescence imaging system (ChemiDoc™ XRS+), fluorescence polymerase chain reaction (PCR) instrument (CFX Connect™ Real-Time), “Quantity One” software (Bio-Rad Laboratories, Shanghai, China); microscope (CX43), fluorescence microscope (CKX53) (Olympus Corp., Shinjuku, Tokyo, Japan); microtome (BQ-318D, Bona Medical Technology, Hubei, China) are used.

### 2.3. Tooth Movement Pain Modeling

After weighing, SD rats were injected intraperitoneally with 1% sodium pentobarbital 150 mg/kg. The four limbs and head of the rat were fixed, and the upper jaw was exposed.

For experimental molar treatment, a 0.5 m deep groove was ground in the neck near to the left upper first molar of the rat with a dental drill. A 0.2 mm orthodontic ligature wire was inserted into the space between the left upper first molar and the second molar and went around the neck of the molars and tightly tied on the inside, forming a small loop of ligature.

For anchorage teeth treatment, a continuous groove of about 0.3 mm was ground on the labial lingual surface of 2 central incisors and gingival margin of the distal surfaces, and the two central incisors were ligated as a whole with the ligature wire.

For NiTi spring fixation, the NiTi spring was tied on the reserved small loop with the ligation wire. When the force was applied, the spring was stretched. The force value was measured with a dynamometer and treatment was performed according to the appropriate force. In the sham operation group, the spring was installed without any corrective force.

For reinforced NiTi spring, the spring was reinforced with medical resin.

For postoperative treatment, after the operation, the rats were kept and fed in the cage. The mouths were examined every day and checked if the spring fell off. Rats in which the spring fell off prematurely were excluded, and one rat was excluded due to the relocated devices.

### 2.4. Experimental Grouping

#### 2.4.1. Experiment 1

For experiment 1, the groups were as follows: (1) healthy control group: no intervention; (2) sham operation group: a force device (NiTi) spring was placed in the mouth, no force was applied; (3) experimental force (20 g) group: a force device (NiTi spring) was placed in the mouth and applied 20 g force, (4) experimental force (40 g) group: a force device (NiTi spring) was placed in the mouth and applied 40 g force; (5) experimental force (60 g) group: a force device (NiTi spring) was placed in the mouth and applied 60 g force; and (6) experimental force (80 g) group: a force device (NiTi spring) was placed in the mouth and applied 80 g force, and the force was applied for 3 days

#### 2.4.2. Experiment 2

For experiment 2, the groups were as follows: (1) healthy control group: no intervention; (2) sham operation group: the force device (NiTi spring) was placed in the mouth for 14 days, and no force was applied; (3) experimental force (3 d) group: the force device (NiTi spring) was placed in the mouth for 3 days and applied 60 g force; (4) experimental force (5 d) group: the force device (NiTi) spring was placed in the mouth for 5 days and applied 60 g force, (5) experimental force (7 d) group: the force device (NiTi spring) was placed in the mouth for 7 days and applied 60 g force; and (6) experimental force (14 d) group: the force device (NiTi spring) was placed in the mouth for 14 days and applied 60 g force

#### 2.4.3. Experiment 3

For experiment 3, the groups were as follows: (1) model+shCOMT adenovirus group: the force device (NiTi spring) was placed in the mouth for 14 days and applied 60 g force. Then, the shCOMT adenovirus was injected into the left infraorbital foramen, and samples were collected 7 days late. (2) Model+COMT antagonist 0.5 mg/kg group: the force device (NiTi spring) was placed in the mouth for 14 days and applied 60 g force. Then, the COMT antagonist 0.5 mg/kg was injected into the left infraorbital foramen, and the samples were collected 7 days later. (3) Model+COMT antagonist 1 mg/kg group: the device (NiTi spring) was placed in the mouth for 14 days and applied 60 g force. Then, the COMT antagonist 1 mg/kg was injected into the left infraorbital foramen, and the samples were collected 7 days later. (4) Model+COMT antagonist 2 mg/kg group: the force device (NiTi spring) was placed in the mouth for 14 days and applied 60 g force. Then, the COMT antagonist 2 mg/kg was injected into the left infraorbital foramen, and the samples were collected 7 days later.

#### 2.4.4. Experiment 4

For experiment 4, the groups were as follows: (1) healthy control group: no intervention. (2) Sham operation group: the force device (NiTi spring) was placed in the mouth for 14 days, and no force was applied. (3) Model group: the force device (NiTi spring) was placed in the mouth for 14 days and applied 60 g force. (4) Model+shCOMT experimental group: the force device (NiTi spring) was placed in the mouth for 14 days and applied 60 g force. Then, the shCOMT adenovirus was injected into the left infraorbital foramen, and the samples were collected 7 days later. (5) Model+shCOMT control group: the force applying device (NiTi spring) was placed in the mouth for 14 days and applied 60 g force. Then, the shCOMT adenovirus control was injected into the left infraorbital foramen, and the samples were collected 7 days later. (6) Model+COMT antagonist 2 mg/kg group: the force device (NiTi spring) was placed in the mouth for 14 days and applied 60 g force. Then, the COMT antagonist 2 mg/kg was injected into the left infraorbital foramen, and the samples were collected 7 days later

### 2.5. Pain Stimulation Test

The rats were kept in the mesh metal cage and acclimatized to the surrounding environment for 10 min. During this period, a small plastic stick was used to repeatedly touch and stimulate the facial whisker pad of the rat at each 30 s interval. When the rat was acclimatized to the surrounding environment and pain was felt as the object approaches the touch, the pain behavior test was started. The test was performed when the rat was in a nonmotion state. During the test, the rat's limbs touched the ground, and there was no movement, shaking, or probing state. Von Frey filaments were used to stimulate the left side of the whisker pad of the rat (ipsilateral to the operation). The stimulation started from 0.008 g and gradually increased, with each stimulation at least 10 s apart until a positive reaction appeared (positive reaction indicator: the rat had the phenomenon of wiping its mouth). The intensity of the stimulation source was recorded. The measurement was performed 3 times continuously at an interval of 10 min, and the mean value of the three measurements was taken as the measured pain threshold.

### 2.6. Pain Behavior Test

In a quiet environment, the rats were placed in a mesh metal cage, and the duration of facial grooming behavior was observed by video recording. The observation method was to place an animal to be observed in a mesh metal cage, and after 15 min of acclimatization to the environment, video recording was started and performed for 10 min each, repeated 3 times, at an interval of 15 min.

### 2.7. Real-Time Fluorescence Quantitative PCR

After RNA extraction, cDNA was synthesized according to the reverse transcription kit. Using cDNA as the template, detection was performed with quantitative fluorescence PCR. With GAPDH as the internal reference, the relative expressions of COMT and IL-17 in each group were calculated. The primers used, operating system, procedure, and analysis were as shown in Tables 1–4.

### 2.8. Western Blot

The trigeminal ganglion tissue of the SD rats was taken and added with the corresponding lysis buffer, lysed at 4°C for 30 min and centrifuged at 10000 rpm/min for 10 min. The supernatant was carefully extracted to obtain the total protein. The bicinchoninic acid (BCA) kit was used for protein concentration determination. Protein denaturation, loading, and electrophoresis were performed for 1-2 h, followed by wet transfer for 30-50 min. Incubation with the primary antibody solution was performed overnight at 4°C, followed by secondary antibody at room temperature (RT) for 1-2 h. Drops of ECL exposure solution were added on the membrane and exposed. The gray value of each antibody band was analyzed with “Quantity One” software.

### 2.9. HE Staining

The tissues were taken out and rinsed with running water for several hours. The specimens were dehydrated with 70%, 80%, and 90% ethanol solutions, followed by equal amount of pure alcohol and xylene mixture for 15 min, xylene I for 15 min, and xylene II for 15 min (until clear). This was then placed in an equal amount of xylene and paraffin mixture for 15 min, followed by paraffin I and paraffin II, each for 50-60 min. The specimens were embedded in paraffin and sectioned. The paraffin sections were baked, dewaxed, and hydrated. The sections which had been placed in the distilled water was then put into the hematoxylin aqueous solution and stained for 3 min. This was followed by hydrochloric acid ethanol differentiation for 15 s, slightly washed with water, blueing for 15 s, rinsed with running water, eosin staining for 3 min, rinsed with running water, dehydrated, cleared, mounted, and examined under microscope.

### 2.10. Measurement of Tooth Movement Distance

A steel ruler was placed on the left maxillary, and photos were taken. The Adobe PostScript (PS) software was used to measure the distance between the disto-, midlingual sulcus of the first molar and the midlingual sulcus of the second molar in the left maxillary of the rats. Measurements were repeated three times, and the mean value was taken, and the orthodontics tooth movement distance was calculated.

### 2.11. FISH Experiment

Paraffin sections were taken out, dewaxed, and hydrated. For the exposure of mRNA nucleic acid fragments, 3% citric acid freshly diluted pepsin (1 ml of 3% citric acid plus 2 drops of concentrated pepsin, and mixed well) was added dropwise to the sections, digested at 37°C for 10 min, and washed with PBS for 5 min (3x) and in distilled water (1x). Fixative solution was added dropwise to the sections, fixed at RT for 10 min, and washed with distilled water (3x). In a humidified box with 20% glycerol, 20 *μ*l of prehybridization solution was added to each section and placed in a 42°C constant temperature incubator for 3 h. The excess liquid was removed without washing. 20 *μ*l/sheet of hybridization solution was added dropwise and incubated at 42°C overnight. 2xSSC at 37°C was washed for 5 min (2x), 0.5xSSC at 37°C was washed for 15 min, and 0.2xSSC at 37°C was washed for 15 min (2x). The blocking solution was added dropwise and placed in a 37°C constant temperature incubator for 30 min. The excess liquid was discarded without washing. 4′,6-diamidino-2-phenylindole (DAPI) was added dropwise and incubated in the dark for 3 min. The nuclei of the specimens were stained. The excess DAPI was rinsed with PBS and with tap water for 1 min. The liquid on the glass slide was absorbed with absorbent paper. The specimen was mounted with antifade mounting media.

### 2.12. Statistical Analysis

SPSS 20.0 software was used for statistical analysis and expressed as mean ± SD. One-way analysis of variance (ANOVA) was used for comparison between groups, and *P* < 0.05 was considered statistically significant.

## 3. Results

### 3.1. Selection of the Appropriate Experimental Force

**Figure 1:**
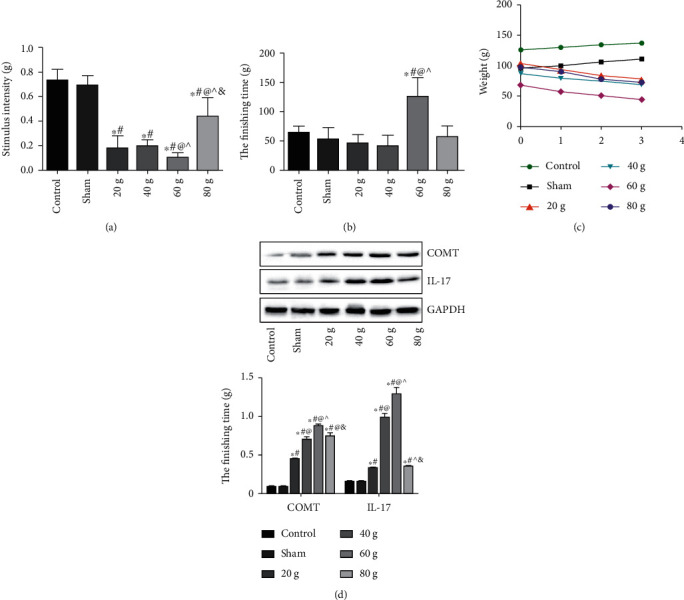
Selection of the appropriate experimental force. (a) Pain stimulation test. (b) Duration of grooming behavior. (c) Weight change of rats. (d) Protein expressions of COMT and IL-17 in trigeminal ganglion tissue of rats. Note: compared with the control group, ^∗^*P* < 0.05; compared with the sham operation group, ^#^*P* < 0.05; compared with experimental force (20 g) group, ^@^*P* < 0.05; compared with the experimental force (40 g) group, ^*P* < 0.05; and compared with the experimental force (60 g) group, ^&^*P* < 0.05.

As shown in Figure 1(a), compared with the control group, the force required for pain stimulation in the experimental force (60 g) group was the lowest (*P* < 0.05). As shown in Figure 1(b), compared with the control group, the duration of grooming behavior of the experimental force (60 g) group was significantly increased (*P* < 0.05). As shown in Figure 1(c), the weight of the rats in the control group and the sham operation group increased steadily each day, while the weight of the rats in the experimental force groups decreased steadily. Among them, the weight loss of the rats in the experimental force (60 g) group was the most severe. As shown in Figure 1(d), compared with the control group, the COMT and IL-17 protein expressions in the experimental force groups were significantly increased, and the expressions in the experimental force (60 g) group were the highest (*P* < 0.05). Therefore, 60 g force was selected for the following experiment.

### 3.2. Selection of the Appropriate Duration for Force Application

**Figure 2:**
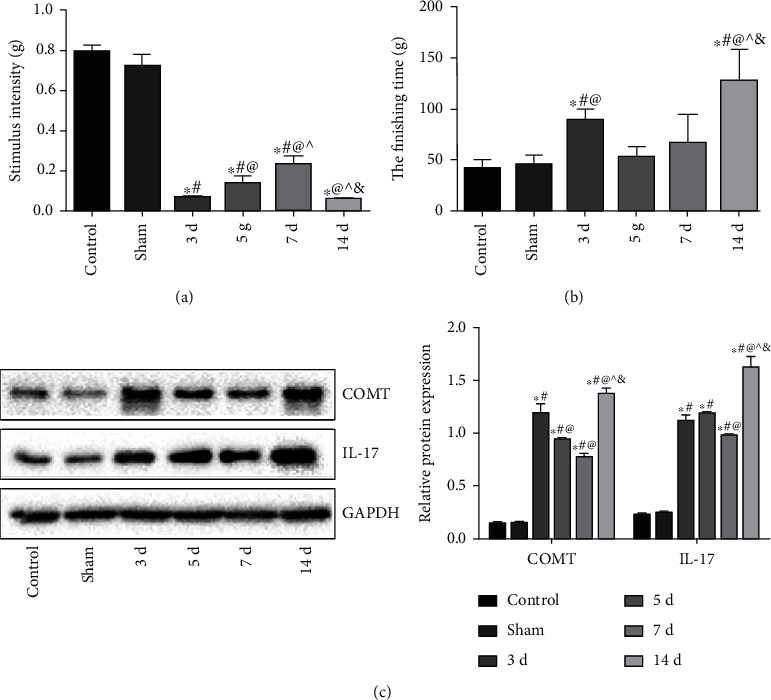
Selection of the appropriate duration for force application. (a) Pain stimulation test. (b) Duration of grooming behavior. (c) Protein expressions of COMT and IL-17 in trigeminal ganglion tissue of rats. Note: compared with the control group, ^∗^*P* < 0.05; compared with the sham operation group, ^#^*P* < 0.05; compared with the experimental force (3 d) group, ^@^*P* < 0.05; compared with the experimental force (5 d) group, ^*P* < 0.05; and compared with the experimental force (7 d) group, ^&^*P* < 0.05.

As shown in Figure 2(a), compared with the control group, the force required for pain stimulation in the 3-day and 14-day groups was the lowest (*P* < 0.05, both). As shown in Figure 2(b), compared with the control group, the duration of grooming behavior of the experimental force (3 d) and (14 d) groups was significantly increased (*P* < 0.05, both), and the duration for grooming behavior of the experimental force (14 d) group was the longest. As shown in Figure 2(c), compared with the control group, the protein expressions of COMT and IL-17 in the experimental force groups were significantly increased (*P* < 0.05 all), and the expressions in the experimental force (14 d) group was the highest (*P* < 0.05). Therefore, 14-day was selected as the appropriate duration for the following experiment.

### 3.3. Selection of the Appropriate COMT Antagonist Concentration

**Figure 3:**
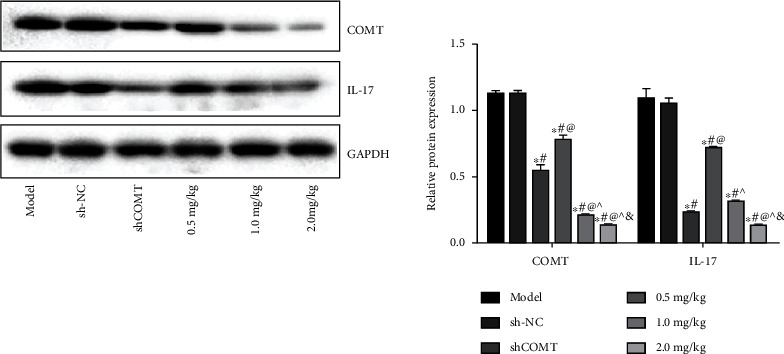
Selection of the appropriate COMT antagonist concentration. Protein expressions of COMT and IL-17 in trigeminal ganglion tissue of rats. Note: compared with the model group, ^∗^*P* < 0.05; compared with the sh-NC group, ^#^*P* < 0.05; compared with the shCOMT group, ^@^*P* < 0.05; compared with the 0.5 mg/kg group, ^*P* < 0.05; and compared with the 1.0 mg/kg group, ^&^*P* < 0.05.

As shown in Figure 3, compared with the model group, the protein expressions of COMT and IL-17 in the shCOMT adenovirus group and the COMT antagonist groups at various concentrations were significantly decreased (*P* < 0.05, all). Among the COMT antagonist groups, the model+COMT antagonist 2 mg/kg group was most significantly decreased. Therefore, 2.0 mg/kg concentration was selected for the following experiment.

### 3.4. HE Staining in Each Group

**Figure 4:**
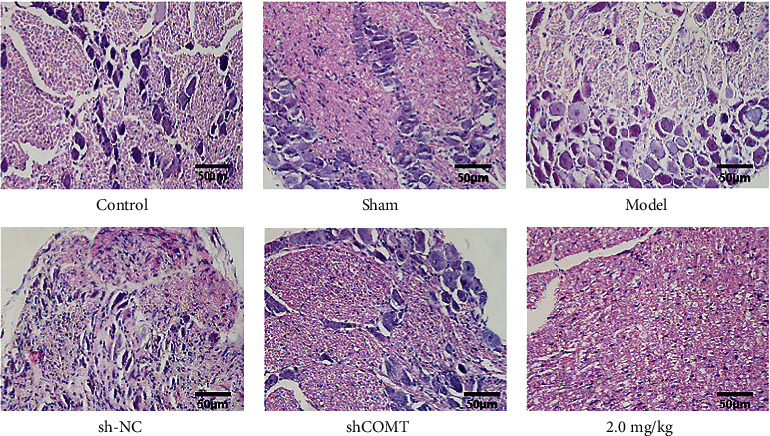
HE staining in each group.

As shown in Figure 4, the control group and the sham operation group have regular morphology; formation of digestive cavity was seen in the model group and the shCOMT control group with a little inflammatory cell infiltration; significant improvement in the injury of the trigeminal ganglion tissue was observed in the shCOMT experimental group and the COMT antagonist group.

### 3.5. Tooth Movement Distance of Each Group

**Figure 5:**
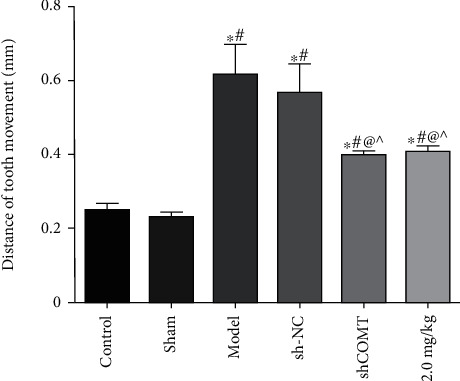
The tooth movement distance of each group. Note: compared with the control group, ^∗^*P* < 0.05; compared with the sham operation group, ^#^*P* < 0.05; compared with the model group, ^@^*P* < 0.05; and compared with the sh-NC group, ^*P* < 0.05.

As shown in Figure 5, compared with the control group, the tooth movement distances of the model group and the shCOMT control group were significantly increased (*P* < 0.05, both). Compared with the model group, the tooth movement distances of the shCOMT experimental group and the COMT antagonist group were significantly decreased (*P* < 0.05, both).

### 3.6. FISH Detection of the Relative Expression of COMT in Each Group

**Figure 6:**
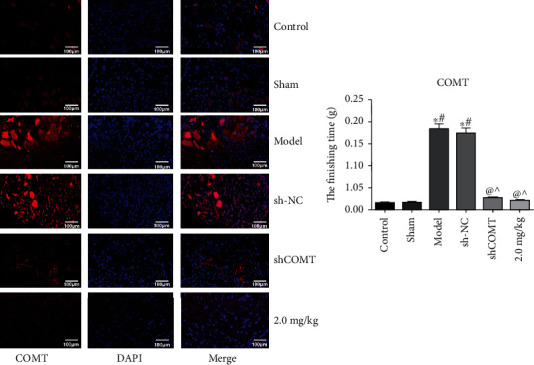
FISH detection of the relative expression of COMT in each group. Note: compared with the control group, ^∗^*P* < 0.05; compared with the sham operation group, ^#^*P* < 0.05; compared with the model group, ^@^*P* < 0.05; and compared with the sh-NC group, ^*P* < 0.05.

As shown in Figure 6, compared with the control group, the relative expressions of COMT in the model group and the shCOMT control group were significantly increased (*P* < 0.05, both). Compared with the model group, the relative expressions of COMT in the shCOMT experimental group and the COMT antagonist group were significantly decreased (*P* < 0.05, both).

### 3.7. COMT and IL-17 Expressions in the Right Trigeminal Ganglion Tissue of Rats

**Figure 7:**
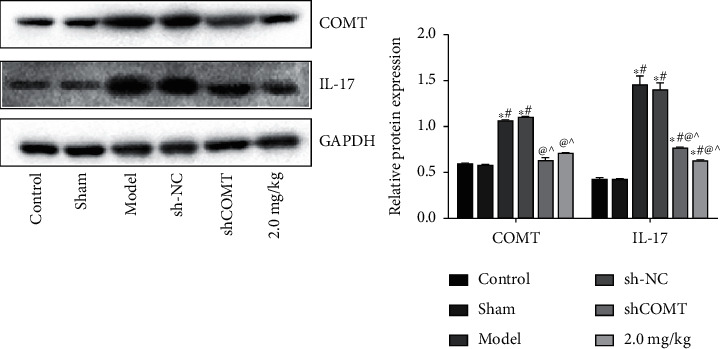
COMT and IL-17 expressions in the right trigeminal ganglion tissue of rats. Note: compared with the control group, ^∗^*P* < 0.05; compared with the sham operation group, ^#^*P* < 0.05; compared with the model group, ^@^*P* < 0.05; and compared the sh-NC group, ^*P* < 0.05).

As shown in Figure 7, compared with the control group, the expressions of COMT and IL-17 in the model group and the shCOMT control group were significantly increased (*P* < 0.05, both). Compared with the model group, the expressions of COMT and IL-17 in the shCOMT experimental group and the COMT antagonist group were significantly decreased (*P* < 0.05, both).

## 4. Discussion

In the process of orthodontic treatment, the most common problems are pain and discomfort caused by mechanical force and the force of the appliance [14–19]. According to the reports, about 90% of the orthodontic patients experience pain during orthodontic treatment [18]. Orthodontic treatment uses mechanical force applied to the teeth to move it, so as to align it and improve occlusion. However, the strength of the appliance can cause local inflammatory reaction, resulting in pain [20]. Behavioral response research is observing the stress response behavior related to the stimulus after applying external stimulus to an organism or individual, thereby is a method of studying the degree of injury and stress response of the body caused by external stimuli. It is widely used in psychology, psychiatry, and sports medicine. In 1998, Yamashiro et al. used grooming behavior to evaluate the pain phenomenon of experimental orthodontic tooth movement in rats, which was recognized by scholars [21]. It made relevant research that is difficult to evaluate in animal experiments to be concrete and quantified and provided a scientific and reliable method for pain research which is difficult to carry out in the past. Later, studies on orthodontic pain, mood, and behavior changes gradually applied this method [22]. Previous studies have shown that directed facial-grooming behavior can be used as an indicator of experimental tooth movement pain behavior in rats [23, 24]. Through the establishment of the experimental tooth movement pain model of rats with different force values and duration, it was found that the 60 g force value and 14-day duration required the lowest stimulus intensity, but the duration of grooming was the longest; the weight loss was the most significant in the experimental force (60 g) group, and the protein expressions of COMT and IL-17 was most significantly upregulated, suggesting that the stimulation intensity and duration of grooming behavior can be used as an indicator to further improve the behavioral evaluation model of pain induced by experimental tooth movement in rats.

The trigeminal ganglion is the portal of nociceptive afferents in the oral and maxillofacial region and an important part of the orthodontic tooth movement pain control [25]. Studies have shown that a variety of pain-related transmitters and receptors exist in the trigeminal ganglion [26, 27]. Studies in local and abroad have shown that the changes of pain-related genes in the trigeminal ganglion are closely related to the occurrence and development of tooth movement pain [28, 29]. COMT is mainly distributed in the postsynaptic neurons and glial cells of the nervous system [30, 31], as well as in the peripheral tissues [32]. In addition, Nicholson et al. found that the COMT gene expression level in the trigeminal ganglion will lead to corresponding changes in the behavioral response of pain in experimental animals [33]. There have been more in-depth studies on the regulation relationship between COMT and pain [34–38], among which are dopamine and epinephrine/norepinephrine neurotransmitters that mainly involved in pain-related regulation [7]. Therefore, we selected COMT antagonist and COMT interfering adenovirus to treat a tooth movement pain mouse model and explored the regulatory role of pain-related shRNA in controlling tooth pain. The results showed that both COMT-shRNA and COMT antagonist injected into the infraorbital foramen can effectively downregulate the COMT gene expression in trigeminal nerve, and the higher the concentration of the COMT antagonist, the more obvious the inhibitory effect.

IL-17 is a proinflammatory cytokine and an early initiation factor of T lymphocyte-mediated inflammatory response. It has a strong proinflammatory effect, and the role of activating and aggregating neutrophils. IL-17 is associated with various bone and cartilage destructive diseases, such as rheumatoid arthritis, osteoarthritis, and periodontitis, and plays an important regulatory role [39]. Studies have shown that IL-17 in GCF may be associated with extensive aggressive periodontitis, and the severity of periodontal destruction and inflammation is concentration dependent [40]. Studies have shown that IL-17 in the gingival crevicular fluid (GCF) may be related to extensive aggressive periodontitis, and the severity of periodontal destruction and inflammation is concentration dependent [40]. IL-17 plays an important role in the regulation of bone metabolism [41, 42]; IL-17 can enhance the activity of osteoclasts, resulting in damage to the bone and cartilage [43]. In the results of this study, IL-17 increased significantly in the model group and decreased significantly with COMT antagonist and COMT-shRNA. Our results showed that the injury of the trigeminal ganglion tissue in was significantly improved, and the tooth movement distance was significantly decreased with COMT antagonist and COMT-shRNA, indicating that COMT antagonist and COMT-shRNA can reduce the pain of orthodontic tooth movement by downregulating the expression level of IL-17. COMT may play its role in the regulation of orthodontic tooth movement pain through IL-17, but the specific path needs to be further studied.

Although *in vivo* experiments with COMT antagonist and COMT-shRNA treatment in orthodontic tooth movement model on detection of their effects on tooth movement distance and IL-17 expression proved that the COMT system has a regulatory effect on orthodontic tooth movement pain, it did not involve the influence of COMT on other factors such as RANKL and OPG or some inflammation-related chemical transmitters and enzymes, and the specific mechanism of COMT had not been studied in detail. Therefore, in the following *in vitro* experiments, we will explore the role of COMT in glial cells and further determine the relationship between COMT metabolism and orthodontic tooth movement pain.

## 5. Conclusions

In summary, by interfering with the COMT gene expression, and thereby affecting the efficiency of tooth movement, the COMT system ultimately has a regulatory effect on the orthodontic tooth movement pain.

## Figures and Tables

**Table 1:** Primer information.

Primer name	Primer name	Primer length (nt)	Product length (bp)	Annealing temperature (°C)
COMT F	CTCCTCCTGCTCTTGCGA	18	237	59.3
COMT R	CATGATTTGGCCTTTCGCGT	20		
IL-17 F	AGTTGGACCACCACATGAATTCT	23	150	60.3
IL-17 R	ACGCATGGCGGACAATAGAG	20		
*β*-Actin F	GCCATGTACGTAGCCATCCA	20	375	59.5
*β*-Actin R	GAACCGCTCATTGCCGATAG	20		

**Table 2:** Operating system.

RNase free dH20	7 *μ*l
cDNA/DNA	1 *μ*l
Upstream primer	1 *μ*l
Downstream primer	1 *μ*l
2x uLtra SYBR mixture	10 *μ*l

**Table 3:** Reaction procedure (3 steps).

Step	Temperature	Time
Predenaturation	95	10 min
Denaturation	95	10 s
Annealing	58	30 s
Extension	72	30 s
Cycle	40	

**Table 4:** Melting curve analysis.

Temperature	Duration
95°C	15 s
58°C	1 min
95°C	15 s
58°C	15 s
58°C	15 s
95°C	0.5°C

## Data Availability

The data used to support the findings of this study are available from the corresponding author upon request.
